# Nursing Roles and Strategies in End-of-Life Decision Making in Acute Care: A Systematic Review of the Literature

**DOI:** 10.1155/2011/527834

**Published:** 2011-10-02

**Authors:** Judith A. Adams, Donald E. Bailey, Ruth A. Anderson, Sharron L. Docherty

**Affiliations:** Duke University School of Nursing, 307 Trent Drive, Durham, NC 27710, USA

## Abstract

The objective of this paper is to analyze the literature concerning nurses' roles and strategies in EOL decision making in acute care environments, synthesize the findings, and identify implications for future research. We conducted searches in CINAHL and PubMed, using a broad range of terms. The 44 articles retained for review had quantitative and qualitative designs and represented ten countries. These articles were entered into a matrix to facilitate examining patterns, themes, and relationships across studies. Three nursing roles emerged from the synthesis of the literature: information broker, supporter, and advocate, each with a set of strategies nurses use to enact the roles. Empirical evidence linking these nursing roles and strategies to patients and family members outcomes is lacking. Understanding how these strategies and activities are effective in helping patients and families make EOL decisions is an area for future research.

## 1. Introduction

End-of-life (EOL) decision making in acute care is complex, involving difficult decisions, such as whether to initiate or discontinue life support, place a feeding tube or a tracheostomy, or initiate cardiopulmonary resuscitation (CPR) in the event of a cardiac arrest. Because of the severity of illness and the nature of treatments, acutely ill patients often lack decision making capacity, which puts the family members in the role of decision-maker [1–4]. One of the biggest challenges to EOL decision making is prognostic uncertainty and determining when to initiate EOL discussions with family members [5, 6]. Nurses and physicians express fear of removing all hope, making the wrong decision or giving up too soon [7–9]. Further, it is difficult emotionally for both family members and health care professionals to give up on curative care [6, 10, 11]. Unfortunately, health care professionals feel inadequately trained to determine when and how to initiate these discussions [6, 12, 13]. Thus, EOL discussions may begin when the physician decides to discuss a do-not-resuscitate (DNR) order, which often takes place when the prognosis is poor [14] and the patient is no longer able to participate. A study of intensive care unit (ICU) cultures revealed that the decision to insert a percutaneous endoscopic gastrostomy (PEG) tube and a tracheostomy often triggered EOL discussions, which, on some units, did not occur until the patient was imminently dying [15]. Another factor that makes EOL decision making difficult is the lack of clarity in the roles of the different health care professionals. Although it is the role of the physician to make a diagnosis and to communicate the diagnosis to the patients or family members, other health care professionals, such as nurses, social workers, and chaplains, have legitimate roles in the EOL decision making process.

The SUPPORT [16] study was a landmark study that set the stage for contemporary research about EOL decision making. Phase I of the SUPPORT [16] study, a multisite investigation into the delivery of EOL care, demonstrated that poor communication about EOL issues resulted in many patients receiving life-sustaining care that they did not want and experiencing moderate-to-severe pain at the end of life. Phase II of the SUPPORT study [16] involved a comprehensive approach to improve the medical decision making and outcomes for critically ill patients, including a nurse intervention. In addition to gathering information from patients and/or surrogates, the SUPPORT nurses provided both educational and emotional support to the patients and family members [13]. Although the intervention arm did not improve the study outcomes of decreasing the length of time to obtain a DNR order, length of stay in ICU, and resource utilization [16], subsequent analyses of narrative data from this study demonstrated that nurses played a significant role in supporting the patients and families and guiding them toward a readiness to withhold or withdraw care [11, 13].

Nurses are at the bedside during the dying process; they spend entire shifts with patients and families, they develop trusting relationships, and they are competent to assess patient and family needs [1, 17]. Nurses gain a unique perspective that allows them to become aware when a patient is not responding to treatment [18, 19]. This perspective places nurses in a position to facilitate EOL decision making. A systematic understanding of what roles nurses enact and what strategies they use in EOL decision making is necessary to ensure that decisions made are consistent with the patient's and family's goals of care.

## 2. Aims

To better understand how to improve the process of EOL decision making, we reviewed the literature to describe how nurses are engaging in EOL decision making with family members of acutely ill hospitalized patients, including the roles that nurses enact and the strategies that they use to enact these roles, as well as the outcomes for patients and family members. The results provide a foundation to improve that process. The purpose of this paper, therefore, is to synthesize what is known about the roles nurses enact, the strategies they use to enact these roles, and how patients and family members respond to these strategies in EOL decision making in acute care settings and to identify questions for significant future research. The findings of the literature paper are organized around the three nursing roles that emerged from the paper: information broker, supporter, and advocate, followed by descriptions of the strategies that nurses use to enact these roles. We present the existing evidence of the outcomes of nurses' engagement in these roles with families and offer recommendations to inform future research.

## 3. Review Methodology

This paper of the literature covers a 15-year period from 1996 to 2011, to capture literature published following the SUPPORT [16] study, a seminal work on this topic. We searched both PubMed and CINAHL using two sets of search terms. Search 1 terms were nurses' role or nursing role or role of nurse PLUS families or family member or surrogate PLUS acute care or hospital care PLUS palliative care or end of life care or end-of-life care or terminal care or withholding or withdrawal or prognosis or quality of life. Search 2 terms were nurses' role or nursing role or role of nurse PLUS families or family member or surrogate PLUS acute care or hospital care PLUS decision making or judgment or resuscitation orders or patient participation. Searches were limited to the English language.

**Figure 1:**
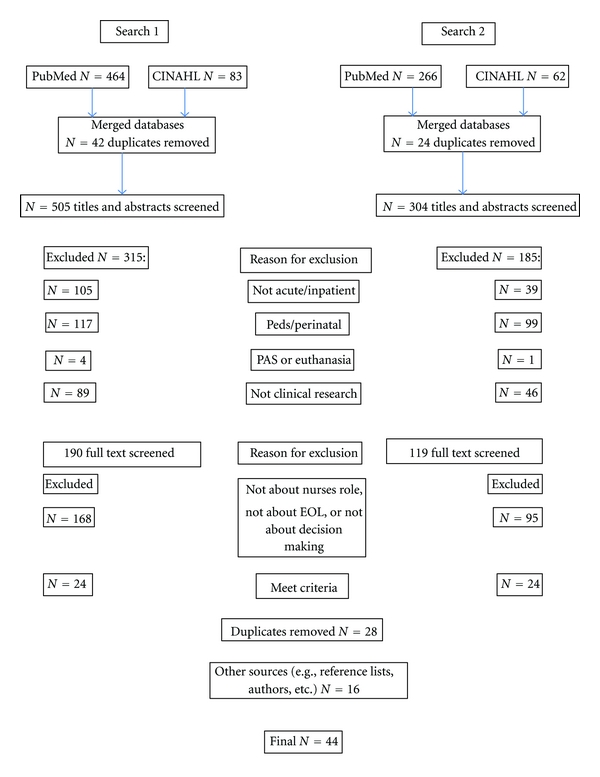
Flow diagram.

As summarized in the flow chart (Figure 1), for the search of each database, after removing duplicates, we reviewed the abstracts and titles for relevance and removed any that were pediatric or perinatal, outpatient/nursing home/or emergency room, not research (i.e., commentaries, case reports, editorials, position papers, and scholarly discussions) or were specifically about physician-assisted suicide (PAS) or euthanasia. Those that remained were scrutinized in more detail by scanning the entire article. We removed any articles that were not relevant to the role of the staff nurse in EOL decision making, excluding articles that discussed EOL care but not decision making, those that discussed only moral distress of nurses, those that discussed the role of the physician or other health care provider but not that of the nurse, and other articles that discussed nurses' involvement in decision making but were not EOL. We also excluded articles that did not address decision making with the family members or surrogates. Although there are some differences in acuity of care and the level of technology between intensive care and acute care hospital units, the findings in studies of nurses' involvement in EOL decision making in these environments were similar enough that we decided to include all studies of inpatient, acute care settings that addressed how nurses are involved in EOL decision making. At the end of this search process, we had a total of 28 articles. We then reviewed the bibliographies of all of the retrieved articles using a snowballing technique that returned 16 additional articles for the paper. 

Forty-four articles identified as meeting our criteria for inclusion were abstracted into a matrix for paper and synthesis. We read each article thoroughly and systematically abstracted its content into a matrix [20] with 11 headings: journal identification, purpose, variables, methodological design, sample design, number of subjects, analysis, validity and reliability, results, significance, and limitations. The matrix helped us examine the literature for patterns and themes across studies as well as relationships among studies. Using an inductive approach, we analyzed the content of the findings and grouped them into four mutually exclusive categories. The designs of the articles were as follows: 32 qualitative, 7 quantitative descriptive correlational, two literature paper, and three mixed methods design. Although 10 countries were represented, only one was multinational [21]. The healthcare professionals who participated in the studies were mainly nurses; however, several of the studies were multidisciplinary including physicians [15, 22–26] as well as social workers, chaplains, pharmacists, ethicists, and respiratory therapists [15, 24]. Six studies included the perspective of family members [23, 27–31].  Table 1 summarizes the study designs, setting, number of participants, participant characteristics, and results.

## 4. Findings

Synthesis of the reviewed literature revealed that nurses' role in EOL decision making has evolved since the SUPPORT study [16]. Results of studies from 1996 to 2000 indicate that nurses were more indirect in their approach. A much-cited study by Viney [26] indicated that nurses perceived that they did not have a legitimate role in EOL decision making. Some nurses reported use of game playing and indirect techniques with physicians to influence decisions [26]. In this and other studies, nurses advocated for patients or family members by informing physicians of patients' expressed wishes and speaking to the physician on behalf of the family [23, 26, 32]. Although there is evidence that some nurses continue to use indirect strategies, more recent studies demonstrate that many nurses now use more direct approaches, such as talking to physicians and family members about prognosis and implications of decisions. This paper also revealed a paucity of evidence examining relationships between nurses' involvement in EOL decision making and patient and family outcomes. From this paper, we found that nurses enact three roles in EOL decision making: information broker, supporter, and advocate. Each role is presented below with a discussion of the strategies nurses use to enact these roles (summarized in Table 2). Finally, we discuss the evidence of the effect that nurses' actions have on patient and family outcomes.

### 4.1. Information Broker

Nurses play an important role in facilitating communication between and among family members and between family members and the health care team (team). The strategies nurses use to enact this role are presented in three categories: give information to physicians, give information to family members, and mediate.

#### 4.1.1. Give Information to Physicians

In a literature paper on the role of the interdisciplinary team in caring for dying patients in an ICU, Baggs et al. [33] found that nurses were described as “information brokers” (page 532), a traditional role described by Viney [26] in which they relay information about the patients and families to physicians. Other studies describe nurses enacting this role by providing the team with information about the patient's clinical status [18, 23], about the patient's and family's emotional and psychological state [25], and about patients' and families' expressed wishes [9, 18, 34]. Liaschenko et al. [8] extended this role further by describing nurses as “nodal points for exchange of information” (page 227) with nurses obtaining information from many sources, synthesizing that information, and using it to develop a holistic assessment. This holistic or “big picture” (page 224) assessment allows nurses to expand their role from information broker to supporter and advocate. These studies demonstrate that nurses are an important source of information to aid physicians in EOL decision making.

#### 4.1.2. Give Information to Family Members

These strategies range from explaining equipment [27], the patient's condition, and diagnosis to the family [13, 24, 27, 35, 36]; translating medical information into lay terms [8, 37, 38]; and explaining, educating, and clarifying meanings of terms, such as DNR [32, 39, 40]. In one study, family members reported that when nurses gave just technical information without synthesizing and interpreting the meaning of that information, they might have instilled false hope [31]. For example, in a neurological ICU, a family member shared that when a nurse told her that the patient's intracranial pressure (ICP) had come down, she was elated, believing that this was a sign that the patient was getting better. In reality, this patient's condition was steadily declining, and the ICP reading was insignificant to the prognosis [31]. Nurses who feel inadequately prepared to give information to family members may give them meaningless information, such as “he/she has had a good night,” [41, page 140]. Family members, in a study of their experience in an ICU, reported that the nurses were often vague, reporting everyday details but not information about prognosis [30].

#### 4.1.3. Mediate

In EOL situations, nurses enact the role of information broker by bringing people together to exchange information directly and facilitating communication among family members as well as between the family and the team. Nurses coordinate family meetings [10, 37, 38, 42] where they may act as a third party to mediate [32]. Nurses may also request the presence of other disciplines, including chaplains or social workers, to mediate EOL discussions [28, 35]. Further, nurses may request that a physician speak to a family [43] or prompt a family member in what questions to ask the physician [12].

The evidence is strong that nurses play an active role in brokering information among family members and the health care team to facilitate communication about EOL decision making. Overall, these studies provided rich data describing the role that nurses play and the strategies they use in gathering and delivering information between family members and the medical team. Further, these findings provide evidence that the role of nurses in informing families is important and valued by nurses and by family members. However, one study included prospective observations [41], and no study followed the participants longitudinally over a period of hospitalization to determine if the information needs changed. A prospective longitudinal study would provide data about changing needs of families and how nurses respond to these needs.

### 4.2. Supporter

Nurses enact the supporter role in EOL care by building trusting relationships with family members as they navigate the EOL decision making process and by demonstrating empathy for patients, family members, and physicians.

#### 4.2.1. Build Trust

Nurses provide support to families by taking time to develop trusting relationships. Family members reported that they trusted nurses who introduced themselves to the family, explained equipment, and were willing to talk [28]. Nurses also identified the importance of establishing a rapport with families [12, 35, 44, 45]. Nurses reported taking time to introduce the family to the oncoming shift nurse to show confidence in that nurse and facilitate the shift change [46]. In this same study, nurses reported allowing family members to take part in daily care and important rituals as a way of supporting the family [46]. Other ways that nurses supported family members included finding out what is important to them, storytelling, life paper, assessing readiness [13], helping with practical needs [18], helping them to maintain a sense of hope [47], accepting their decisions [32, 43], and preparing them for bad news [18].

#### 4.2.2. Empathy

The literature is replete with examples of nurses enacting the role of supporter to family members of patients at the EOL through the use of empathy, using strategies such as trying to understand how the family members see the situation [18], being present [7, 11, 13, 39], taking time to listen, allowing the family time to process the information given [11, 39, 43, 48], and acknowledging feelings [13, 26, 29, 37]. In one study nurses expressed a desire to support their physician colleagues by being “someone physicians can talk to” (page 165), so that the physicians would not feel alone in the decision making [9].

Most of these studies were descriptive in nature and focused on the perspective of nurses and/or physicians and their perception of the role of nurses in EOL decision making. Few included the perspective of the family members. In addition, most of the data were retrospective in nature, gathered from interviews, focus groups, or analyses of narratives where nurses described their own perception of their involvement and how families responded to this involvement, without including input from the family. 

The literature provides evidence that nurses enact the role of supporter by use of many strategies to build trust and demonstrate empathy during the end of life period and that family members value this support, but the evidence of whether or not family members find this support helpful in the decision making process is lacking. Prospective studies that include the perspective of family members and nurses along with observations of interactions would provide evidence of the support that nurses are actually giving and how family members respond to that support.

### 4.3. Advocate

Understanding about nurses' role as patient advocates at the EOL has evolved from indirect to active. The enactment of the advocate role may take the form of speaking to the medical team on the behalf of the patient or family as well as speaking to the family on behalf of the patient. One strategy that nurses use in both situations is to challenge the status quo. The expectation of family members and health care professionals in acute care is often one of cure with a tendency to pursue aggressive treatments that may be futile. In an attempt to advocate for their patients, nurses often find themselves in a position of challenging physicians and family members to consider changing the direction of care from curative to palliative.

#### 4.3.1. Advocate to Physicians

“A voice to speak up” (page 504) was a theme that emerged from a grounded theory study of nursing roles in EOL decision making in the ICU [39]. In this and other studies nurses reported several modes of advocating, including going directly to the physician to report the expressed wishes of the family and questioning physicians about the plan of care [9, 39]. In another grounded theory study of nurses' roles in withdrawal of life-sustaining treatment nurses described a more indirect mode of dropping hints or “planting the seed” (page 254) to physicians that it may be time to change from curative to palliative care [18]. In other studies nurses have described coaching physicians [43] and timing EOL discussions to coincide with the schedule of a physician most likely to be open to EOL discussions [15]. 

Some of the studies described a more assertive nursing role with nurses reporting pushing physicians to change the direction of care [49], using results of prognostic tools to discuss the patient's prognosis with the physician [50], and arguing with physicians about the plan of care [36]. In a study of expert nurses in critical care, a nurse described challenging a physician in front of the patient when the nurse perceived that the physician was not being honest with the patient; another nurse refused to carry out orders that were against the patient's expressed wishes [35].

#### 4.3.2. Advocate to Family

The literature provides evidence that nurses advocate to families on behalf of patients about EOL decision making. Nurses reported gently informing family members that their loved one was dying [51]. Nurses helped family members to clarify the goals of care, challenging them to consider what the patient would have wanted [35, 39] and explaining the implications of decisions [8, 34, 40]. Nurses facilitated decision making by presenting a realistic picture of what was happening, coaching the family members to make decisions that were consistent with their goals, [35, 40] and helping them to accept the inevitability of death [39]. 

One nurse emphasized the importance of explaining the implications of the diagnosis, stating “they may not have agreed…if they'd known all that” [39] (page 506). Nurses recognize that when a patient goes on life support, the result may be to prolong the dying process. In a study of EOL communication in the ICU, two nurses eloquently described the dilemma in which family members find themselves when patients opt for aggressive treatment that they may not comprehend [8]. The first nurse in this study described why some patients change their mind about intubation when in distress. “Because they are scared…They reverse their decision because the doctors ask them without communicating the whole picture” (page 227). The second nurse described her conversation with a patient thusly, “I said, “he's asking you if you want to go on life support or if you want medication to keep you comfortable so you're not scared (while dying)” When I clarified this for the patient he chose option 2” (page 227). 

In the study by Robichaux and Clark [35], expert nurses described taking a very assertive role in the EOL decision making process with families. One nurse explained to a daughter that the ventilator was not helping her mother with end stage COPD to feel better or breathe easier, explaining that, in fact, it was difficult to be on a ventilator. This daughter did eventually agree to withdraw the ventilator, and the patient died peacefully [35]. Other nurses in this study had the family members participate in daily care, such as suctioning and turning to allow the family member to see the decubiti, in an attempt to show the family member possible physical discomfort that the patient was experiencing. One nurse told a family member, “we're torturing him” (page 487). 

Liaschenko et al. [8] found that nurses synthesized information to obtain a holistic view and gently challenged family members to consider the consequences of continued aggressive care. Nurses used the fact that patients were not responding to treatment or were continuing to deteriorate to facilitate having these discussions. In a study of the phenomenon of transition from curative to palliative care, nurses described cueing the families about changes in the patient's condition that may indicate deterioration and a need to change course [49].

Family meetings provided a forum for nurses to advocate for patients and family members. Nurses described speaking out in family meetings by expressing their opinions and the wants and needs of patients and family members, listening, and clarifying information [39]. Hsieh and Shannon [22] found that nurses were present in 41 of 50 family meetings that were recorded, and some nurses were actively involved. One nurse spoke eloquently explaining to the family members the expressed wishes of a patient not to be intubated, his agreement for a trial of a few days, and his wish to be taken off the ventilator after that time; this meeting took place six weeks after the patient's expressed wishes [22]. Another nurse asked a family, “If he could sit up right now, what would he say to you. Would he say he wants to go on with all this? Would he say, stop, that's enough” [22] (page 301)? Although Sorensen and Iedema [45] found that nurses were not routinely included in family meetings and were not privy to what physicians had told the family, one nurse expressed that when attending a family meeting he/she would “put in my five cents' worth…at the end of it” (page 191). 

Interviews with family members of patients who died in ICUs revealed that family members had expectations that nurses would enact the role of advocate by providing meaningful information about patient prognosis [27]. In other studies, family members expected nurses to give honest information about how the patient's condition was progressing as well as an interpretation of that information [28, 31]. The participants in the study by Verhaeghe et al. [31] reported that nurses sometimes gave them only facts without interpretation, which lead to confusion and misunderstanding. Surrogates of patients who had died after withdrawal of life support in an ICU reported that nurses provided information to them about the condition of the patient and helped them to understand futility [29]. One study of family members revealed that nurses often were not present in family meetings, gave vague information, and did not answer questions directly; however, participants in this same study identified some nurses who were more forthright and gave clear information [30].

#### 4.3.3. Extent of Nursing Advocacy

Estimates varied about the extent to which nurses are currently enacting an advocate role in EOL decision making. Kennard [23] found that nurses advocated for their patients only 53% of the time, whereas Ho et al. [52] found that 78% of ICU nurses were actively involved in EOL decision making, and 42%–54% actively discussed EOL decisions with the patient or family. In a study of ICU and oncology nurses' involvement in DNR orders, 81% reported taking on the role of patient advocate, and seven percent reported taking on the role of decision-maker [53]. A survey of ICU nurses' attitudes about EOL decision making revealed that 95% believed that nurses need to respect patient's wishes, 98% would talk to a physician if a patient's wishes are violated, 96% would help inform the patient/family of the condition and treatment options, 98% counseled the patient/family about advance directives (AD), and 85% initiated discussion of ADs [54]. In a study of nurses' attitudes about EOL discussion 95% of the nurses believed it was their responsibility to talk to physicians' about the patient's living will, but only 50% reported participating in DNA discussions [55]. In a study of ICU nurses' attitudes about withdrawing treatment, 75% of nurses reported they were actively involved, and 64% said they had initiated discussions with the physician [21].

The findings from studies utilizing qualitative designs indicate that nurses perceive that they are challenging physicians and family members to see the big picture, consider the patient's wishes, consider the implications of decisions, and consider changing the direction of care from curative to palliative. The findings also demonstrate that family members have the expectation that nurses should be actively involved in the decision making process by providing prognostic information and that nurses do not consistently do so. Evidence from quantitative studies about the level of nursing advocacy in EOL decision making is sparse and provides conflicting results. 

All but one [15] of the above studies about the role of advocate were retrospective and none included observations of what nurses were actually doing and how these activities affected the decision making process over time. A prospective, longitudinal study combining interviews with observations would allow a comparison of nurses' reports of advocating with observations of these strategies. In addition, these data would describe whether and how decisions are altered by physicians and family members when nurses take an active role to advocate for a change in the direction of care from curative to palliative.

### 4.4. Patient and Family Outcomes

Nurses recognize their potential effect on EOL decision making [8], yet few studies addressed this effect. Although there is little empirical evidence of the effect nurses have on patient and family outcomes, the literature suggests that the roles and strategies nurses enact do affect the family members' ability to accept that the patient is dying and do affect the overall decision making process. In a literature review, Frank [48] asserted that, through their roles in facilitating communication and allowing patients to remain in control, nurses can increase the likelihood of a good death.

#### 4.4.1. Accept That Patient Is Dying

Several qualitative studies indicated that nurses believe that the strategies they use help family members accept that a patient is dying by bringing families to “readiness” [13], “enabling coming to terms,” and “helping to let go” [39]. Increased involvement of nurses in shared decision making helped family members in one study to understand and accept the prognosis and to prepare for and cope with death [30]. In a study of family members of patients suffering from traumatic coma the participants expressed that the way they received information affected their hope and that when they received incomplete information or just facts, they were likely to misinterpret the information and have unrealistic hope [31].

#### 4.4.2. Making Better Decisions

To make decisions, family members need to understand the condition of the patient and the options available; this necessitates receiving clear and truthful information. Studies of perceived needs of family members demonstrate that when family members developed trusting relationships with nurses, they could ask nurses questions, trust that they would get the truth, had a better understanding of the prognosis, and were more prepared [28, 30]. In addition, one study showed that when family members' needs were met, they were more satisfied with the care [27]. A grounded theory study of EOL decision making revealed that family members' relationships with the nurses helped to move them along in the decision making process [29]. 

Studies of nurses demonstrate that nurses believe that what they do is important in preparing the family [8] and moving the family along in the process of decision making [11]. Nurses also express that when there is a smooth transition from curative to palliative care, they are better able to manage pain and symptoms [49]. Some nurses expressed a fear that involving family members in EOL decision making would lead to a sense of burden or guilt on the part of the family member [9, 13]. One nurse expressed his/her concern that family members may feel like they “held the patient's life in their hands” [9] (page 168). This sense of burden could affect a family member's ability to make EOL decisions.

Evidence from studies of family members suggests that family members find nurses' involvement to be sometimes beneficial and at other times harmful, such as when nurses give information without interpretation leading to false hope. In an analysis of data from the SUPPORT [16] study, researchers found that over half of the subjects reported that nurses' involvement was helpful [23]. With the exception of two [23, 27], the studies of family members or surrogates were qualitative and contained thick descriptions of family members' experiences with nurses. The two quantitative studies used measurement tools that were developed based on information from nurses rather than information from the family members. The data suggest that family members may benefit from nurses' taking an active role in providing meaningful information about patient prognosis.

Nurses believe that families benefit fromthe strategies they use to enact the roles of information broker, supporter, and advocate although some fear that family members carry a heavy burden of decision making. In addition, families have reported that they found some of the strategies used by nurses to be helpful, especially when family members develop trusting relationships with nurses, receive adequate truthful information, and engage in shared decision making. There is, however, no empirical link between specific nursing roles and strategies and outcomes for the family members of patients in acute care at the end of life. Further, there is a lack of evidence to explain how and why the roles that nurses enact are important to patients and family members.

## 5. Discussion

Since the findings of the SUPPORT study were reported in 1995, there has been a focus in the literature on improving communication that facilitates decision making between physicians and patients/families at the end of life, with little focus on the role of nurses in EOL decision making processes [1]. In those studies where nurses and physicians collaborated about EOL decision making, positive outcomes, such as decrease in LOS, were achieved [56–58]. Yet these studies were few, lacked experimental control and internal validity, and did not adequately describe the nurses' role, nor did they attempt to measure family member outcomes. 

The literature describing how nurses are involved in EOL decision making can be summarized by the enactment of three nursing roles. First, as information brokers, nurses provide information about the patient and family to the health care team, provide information to the family about the patient, and coordinate EOL discussions. Secondly, as supporters, nurses provide an important source of emotional support to family members as they process the information they are given and attempt to make decisions. These forms of support include building trust and empathy. Thirdly, and most importantly, whereas earlier literature indicated that nurses were involved in an indirect manner, recent literature indicates that nurses are more actively engaged as advocates in EOL decision making with both physicians and family members, challenging the status quo and helping all of the parties to see the big picture. Further, the literature suggests that when nurses are actively engaged with family members by interpreting and explaining to them what is happening and explaining prognoses, family members are more satisfied and able to move forward in their acceptance and decision making.

### 5.1. Overview of Strength of Evidence

The literature regarding nurses' involvement in EOL decision making is based mainly on qualitative designs and provides rich data with thick descriptions of the experiences of nurses and family members in acute care end of life situations. Most of the authors provided evidence of qualitative rigor, specifically confirmability through use of audit trails, dependability through use of multiple investigators in coding and analysis, credibility through use of search for disconfirming evidence and triangulation of data, and transferability through rich descriptions of findings. Four qualitative studies did not address or demonstrate qualitative rigor [34, 37, 49, 51]. Two studies mentioned methods of maintaining rigor, such as credibility, confirmability, and auditability, but did not describe how this was done; both of these studies demonstrated transferability through rich descriptions [13, 42]. 

Seven quantitative studies described the level of involvement of nurses in EOL decision making and the expectations of involvement by nurses and family members. Four of these studies provided no psychometrics for the instruments used [21, 23, 52, 55], and three did not address the content validity of the instruments [23, 52, 55]. In their study of family members' perceptions of nurses' role, Fox-Wasylyshyn et al. [27] established content validity from nurses rather than from the family members, who were the stakeholders. The content validity ideally should have come from the literature or from qualitative research of family members. The study by Scherer et al. [54] had only a 21% return rate leaving the findings vulnerable to selection bias. Overall the findings from the quantitative studies lacked validity.

The literature suggests that what nurses do is helpful to patients and families; there is limited empirical evidence, however, to demonstrate the unique and important role nurses have in EOL decision making. Further, there is little evidence that patients and family members who have experienced an actively involved nurse will fare better than those who did not, and there is lack of a clear definition of what it means for a nurse to be actively involved in EOL decision making.

## 6. Implications for Nursing Education, Practice, and Research

As the global population ages and technology permits prolongation of life, dealing with dying patients and their families is likely to become a more frequent experience for all nurses, especially those in acute care environments. Nurses need guidance in enacting their roles in EOL decision making so that they can alleviate suffering and ensure that EOL needs and goals for their patients and family members are being met. These nursing strategies should be guided by empirical evidence. Knowledge of what strategies nurses use to enact their roles and what strategies are beneficial to patients and family members will guide nursing education, practice, and research. Understanding how nurses engage in this process is essential to the development of interventions to improve the strategies that nurses use in EOL decision making. 

Prospective, longitudinal, and case-oriented studies that identify how nurses engage in the EOL decision making process and how the engagement changes with the needs of the family over the time of the hospitalization and explore the effect that nursing interventions have on patient and family member outcomes are needed. Such studies would provide a systematic understanding of the strategies that nurses use and how and why family members respond to these strategies. In addition, strong quantitative studies that build on the rich descriptions in the existing literature to develop valid and reliable tools are needed to measure the extent to which nurses are enacting roles, the strategies they use, and the patient and family member outcomes. This would pave the way for larger-scale quantitative studies and provide ways to assess any randomized controlled trials. With the knowledge generated from these studies, interventions could be developed that target areas identified as important to the family members and most likely to improve their well-being. This knowledge would allow for spreading expert nursing practices to all nurses in a systematic fashion, helping family members make decisions that are consistent with their values and goals for EOL care and reducing the amount of psychological distress of family members who make EOL decisions in acute care environments.

## 7. Conclusions

This paper highlights the important role of nurses in EOL decision making. Although nurses believe that their involvement is beneficial to patients and family members, this paper reinforces the need for empirical evidence of these benefits at the end of life, especially as it relates to the well-being and coping of family members who are making difficult decisions about a loved one in an acute care environment. Better understanding of how nurses enact their roles in EOL care could improve the overall quality of communication in EOL care and help more patients and families make decisions that are consistent with their values and goals for EOL care.

##  Authors' Contributions

All four of the authors contributed to the conception and design of the paper. J. Adams undertook data collection, analysis, synthesis, and drafting of the paper. She is a Ph.D. student at Duke University. Jr. D. Bailey, R. Anderson, and S. Docherty reviewed and critiqued the initial synthesis, D. Bailey and R. Anderson reviewed and critiqued the subsequent drafts. All authors have critically reviewed and approved the final paper.

##  Conflict of Interests

The authors declare that there is no conflict of interests.

##  Funding

The following sources provided funding for the research of the primary author but have no role in the design, collection, analysis, interpretation, reporting, or the decision to submit for publication: Duke Translational Medical Institute: Clinical Translation and Science Awards, National Institute of Health Grant no. TL1RR024126 and Duke University School of Nursing Ph.D. Fellowship. Hospice and Palliative Nurses Foundation.

## Figures and Tables

**Table 1:** Summary of findings.

Author	Research design	Setting/sample	Themes
Bach et al. [39]	Grounded theory	2 Critical care units in teaching hospital; 14 nurses; Canada (Ontario); 14 nurses	Supporter: be present with families and listen. Advocate: help family to understand the implications of decisions, question physicians, speak up and give opinions at family meetings, and help family think about what patient would want. Initiate discussion with physicians, explain things to family in lay terms, and give honest information without taking away hope. Outcomes: “Enabling coming to terms” and “helping to let go.”

Baggs et al. [33]	Literature paper	ICU; US	Information broker: nurse as information broker and mediator. Outcomes: decrease costs and LOS and improved communication with multidisciplinary/collaborative interventions.

Baggs et al. [15]	Ethnographic	ICU; 34 case studies; US	Advocate: nurses timed EOL discussions for when a physician was on rotation who was seen as open to discussing EOL issues.

Barthow et al. [40]	Qualitative descriptive	Tertiary cancer center; 21 nurses; New Zealand	Information broker: provide and clarify information. Supporter: coaching, facilitating, and offering choices. Advocate: help clarify goals and help family to understand ramifications of decisions.

Bushinski and Cummings [37]	Qualitative" “appreciative inquiry” (Hammond).	Outpatient palliative care and a MICU; 8 nurses; US (Minnesota)	Information broker: interpret what physician said. Arrange for family meetings. Supporter: build trust, acknowledge emotions, explore statements, pause, allow time, be present recognize cues of readiness to talk, support, sit close and make eye contact, turn off phone and beeper, do not look at watch, rephrase, and explore emotions. Advocate: ask leading questions.
Calvin et al. [38]	Qualitative descriptive	Neuro ICU; 12 nurses; US	Information broker: translate medical terms. Suggest and set up care conferences Supporter: listen, maintain close connection with family, reassure. Advocate: elicit values, thoughts, and understandings from families, push family to make decisions at times, try to get MD to see big picture. “If she's not getting better, not waking up, then that's a sign that her brain is not functioning...and that should tell you that you need to take mom home and you need to love her and make those last days of her life more comfortable than being poked or prodded.” page 147

Calvin et al. [44]	Qualitative descriptive	CVICU; 19 nurses; US	Advocate: Acknowledge physician authority, and walk a fine line. Try to prepare family member try to tell families without really telling them. Some would tell family even at risk of being reprimanded Supporter: Promote family presence.

Engstrom and Soderberg [47]	Qualitative: focus groups	ICU; 24 nurses; Sweden	Supporter: nurses felt it was important to maintain hope and not give false hope. Balance hope with realism. Hope for good death. Advocate: difficulty being honest when given conflicting info from physician and when doing treatments nurse disagrees with.

Espinosa et al. [12]	Descriptive phenomenological	ICU; 18 nurses; US	Information broker: tell family members what they need to ask the physician. Supporter: build a trusting relationship with families.

Fox-Wasylyshyn et al. [27]	Descriptive correlational	ICU; 29 family members; Canada	Information broker: explain equipment. Advocate: explain prognosis. Outcomes: increased satisfaction with care.

Frank [48]	Literature paper	Acute care and hospice settings; 9 articles; UK	Information broker: communicate honestly Supporter: allow patient time to make decision, support patient, recommendations of how nurses should enact their roles, engage in process with patient and physician, and develop trusting relationship based on power sharing. Advocate: Be assertive. Outcomes: assertion that nurses can increase the likelihood of a good death.

Fry and Warren [28]	Phenomenology	ICU; 15 family members with varied ethnic and cultural backgrounds; US	Supporter: build trusting relationships, introduce self to family and explain equipment, and demonstrate openness and willingness to talk. Advocate: give honest information about how patient is responding to treatment. Outcomes: developing trusting relationship allows family to feel that they can ask the nurse about the patient and trust that they will get the truth.

Harris [10]	Grounded theory	ICU; 9 nurses; UK	Advocate: advocated for care conferences.

Haslett [53]	Cross-sectional explanatory descriptive	Acute care; 278 nurses (68% response rate); US	Information broker: educate 68%, give information 58%. Advocate: advocate 81%. Only 7% assumed role of decision-maker (determining whether DNR was appropriate).

Heland [42]	Qualitative descriptive	ICU; 7 nurses; Australia	Information broker: arrange for family meetings, and coordinate the meetings to get all the interested parties together. Advocate: explain the patient's condition to the family.

Hildén and Honkasalo [34]	Qualitative interview	Acute, long-term, and home settings; 17 Nurses; Finland	Information broker: provide information to the physician Supporter: provide emotional and existential support. Advocate: clarify information given by the physician by presenting it in a way that they can understand in lay terms. Help family understand the pros and cons of decisions. Lead the family and help them to see reality.

Hildén et al. [55]	Descriptive: questionnaire	All areas of care; 408 Nurses (51% response rate); Finland	Advocate: 95% nurses felt it was their responsibility to talk to MD about a patient's LW if it was not being respected. 50% reported that they participated in DNR discussions with families when patient unable to communicate.

Hiltunen et al. [11]	Narrative content analysis	5 hospitals; 23 nurse facilitators; US	Supporter: “Midwife-one who understands the process unfolding and can be present, with the family” page 132. Skill, patience, being present and sharing the experience with the family. Outcomes: move the family along in the decision making process.

Ho et al. [52]	Survey	ICU, NICU, peds; 611 nurses; New Zealand	Advocate: 78% of participants said they were “actively involved” in EOL decisions. 42–54% actively discussed EOL issues with patient or family. Actively involved defined as “active discussion with patients, families, or physicians in the decisions to withdraw life support or withhold cardiopulmonary resuscitation.”

Hov et al. [7]	Qualitative: phenomenology	ICU; 14 nurses; Norway	Supporter: presence, see changes, holistic. Advocate: interpret what is going on with the patient and give their interpretation to the physicians “using different strategies” (these strategies not described). Help physician understand the suffering. Go to physician meetings and express their opinion.

Hsieh and Shannon [22]	Qualitative content analysis	ICU; 51 family meetings; US	Advocate: actively participate in family meeting. Relate to family what patient said before becoming unconscious. (very eloquent description of what patient wanted given by a nurse in a family meeting). Ask what pt. would want.

Jezewski and Finnell [32]	Grounded theory	Acute oncology settings; 21 nurses; US (New York)	Information broker: be a third party to mediate among family members or between family and providers. Tell the physician what patient's wishes are. Supporter: be sensitive to family members' emotions. Listen, caring, assess emotional readiness. Advocate: help patient and family understand what DNR means. Be sure they are informed and support their decisions.

Kennard [23]	Descriptive	Acute care; 1427 patients/surrogates, 696 nurses; 5 settings; US	Information broker: 95% reported that they gave information to the medical team about patient's medical status Advocate: on day three, 67% had no knowledge of their patient's preferences. 53% reported not advocating for patient preferences. 17% discussed prognosis with patients, 32% offered recommendations to the family or patient. 58% discussed options and educated about the treatments. Outcomes: 50% of patients or surrogates thought conversations with nurses were “very much” or “quite a bit” helpful in their decision making. 25% felt that nurses preferences had “quite a bit” or “very much” influence on their decision.

Kirchhoff et al. [46]	Cross sectional qualitative descriptive	ICU; 21 nurses; US	Supporter: introduce the nurse coming in on next shift, show confidence in that nurse, facilitate shift change, and show you care. Allow time to accept, facilitate, allow family to participate in care, make time and space for family rituals. Advocate: nurses believed it is physician's responsibility to give family information on prognosis initially. Fear of taking away hope, do not like to see families being given false hope. Families “look to nurse for “real” answer.” page 39

Latour et al. [21]	Descriptive correlational using survey	ICU; 62 nurses; UK, Netherlands, Italy, Norway, Sweden	ADVOCATE: 75% reported active involvement in decision. 39% reported being asked to participate by MD. 64% said they had initiated discussions w MD, 52% said they were not actively involved in discussions w physician colleagues.

Liaschenko et al. [8]	Qualitative: focus group	ICU; 27 nurses; US	Information broker: “Nurses are nodal points for exchange of information” page 227 nurses obtain info from physicians, families and synthesize the info to develop a comprehensive picture of what is happening. Supporter: supporting journey. Build trust. Advocate: helped families see the “big picture,” including QOL and continued deterioration. Tell families about consequences of interventions. “what are the chances of improving their quality of life.” Outcomes: one nurse expressed a belief that they can have a significant impact on the outcomes and the need to be sure they are advocating for the patient.

Limerick [29]	Grounded theory	ICU; 4 hospitals in a system, 17 surrogate decision-makers; US (Texas)	Information broker: provide information Supporter: support, caring, sensitive, build trusting relationships. Advocate: help family member to understand what is happening with the patient and recognize futility. Outcomes: help move family along in the decision making process by helping to build trust and help family member to understand the futility of the situation.

Lind et al. [30]	Grounded theory	ICU; 3 University hospitals, one district hospital, 27 family members; Norway	Information broker: nurse communicated about everyday issues not about prognosis or decision making. Nurses were vague and reluctant to give information. Some reported that the nurse did give information and was clear. Those were the families who reported shared decision making. Nurses rarely involved in family meetings, nurses did not answer questions. Outcomes: shared decision making with increased involvement of nurse seemed to improve family members understanding of prognosis and they were more prepared to cope with death.

McMillen [18]	Grounded theory	ICU; 8 nurses; UK	Information broker: provide medical team with information about families' viewpoint and about clinical status of patient. Supporter: support the family, prepare them for the bad news, find out what is important to them, how they see the situation. Help family with practical needs. Also attend to families' practical needs. Advocate: drop hints to physician. Question physician.

Murphy et al. [13]	Content analysis	5 hospitals; 20 nurses; US	Information broker: educate about the disease process. Facilitate communication between family and staff. Supporter: presence, listening, empathy, explaining, clarifying, storytelling, and life paper, assessing readiness. Advocate: discuss prognosis with family members and expected outcomes of treatment. Outcomes: nurses expressed sense that families were burdened by being offered futile care.

Reckling [24]	Multiple case study.	ICU; 16 family members, 29 health care professionals including 15 Nurses; US	Advocate: nurses did not participate in the initial discussions about withdrawal but did talk to families once the physician had brought it up. Of the 15 nurses observed, only 3 took a strong advocate role, the other 12 were either moderate advocate or neutral.

Robichaux and Clark [35]	Qualitative: narrative analysis	ICU; 21 nurses; US	Information broker: educate, consult other services, such as requesting an ethics consult. Supporter: establish trust, assess when is the right time to initiate discussions. Advocate: advocate, speak up for patient even if it risks being reprimanded. One nurse spoke in front of family when physician was not being honest. One refused to carry out orders that were against patients expressed wishes. Help family to reframe their hope. Speaking to family on patient's behalf. Help family to understand the situation, for example, ventilator does not help a person feel better. Show family what it is like (let them see suctioning, decubiti, etc.). One nurse told a mother, “we're torturing him.” page 487

Scherer et al. [54]	Descriptive correlational using survey	ICU; 210 nurses (21% response rate); US	Advocate: 96% had helped inform patients or families of condition and treatment options. 98% counseled patients or families about AD, 85% initiated discussion of AD.

Silén et al. [9]	Qualitative content analysis	Dialysis units and nephrology wards; 13 nurses; Sweden	Supporter: support physicians, be available for patients and physicians to talk to. Advocate: question physicians while at the same time recognizing the difficulty of the physicians' position. Information broker: convey information, for example, tell physician about patient wishes and any questions that families have raised. Outcomes: belief that the family may be burdened by the responsibility and feel they “held the patient's life in their hands.” page 168.

Sorensen and Iedema [45]	Grounded theory	ICU; 30 nurses; Australia	Information broker: nurse feels caught between family and physician. Family wants to talk to physician, nurse asks physician to talk to family, and physician says he/she has already talked to them Supporter: let family express their feelings, be sensitive to feelings. Establish a rapport, prepare families. Advocate: some of the nurses in study did not advocate for patient and did not give any professional opinion to the physician about the appropriateness of continuing aggressive care. Others talked to MD and told them of patient's preference to stop treatment. Nurse expressed putting in his/her “five cent's worth” at the family meeting. Nurses often left out of meetings. Importance of ongoing discussions of the plan of care and what the next steps would be if treatment does not work.

Sorensen and Iedema [25]	Ethnographic	ICU; Tertiary care hospital, 13 case studies, 15 family conferences, 29 focus groups with nurses, interviews with medical and nursing management; Australia (Syndey)	Information broker: provide information about patients' emotional and psychological status. Nurses contribute knowledge about psych and emotional issues to the team. What seemed to be missing was “a therapeutic engagement” of the nurse with the patient and family. Advocate: nurses see suffering but may be reluctant to speak frankly to family.

Thompson et al. [49]	Grounded theory	Acute care; 2 hospitals, 10 nurses; Canada	Information broker: empower by giving information, mediate, clarify information given by MD. Advocate: assess what patient/family understand, educate about disease process and possible outcomes, communicate honestly, cue patient/family and physician about signs of poor prognosis, push for DNR order from MD, encourage family to consider what pt. would want. Outcomes: “Smooth lane change” leads to collaborative care plan, appropriate level of care, ability to address symptoms, psychological support. Failure to do so leads to false hope for patient/family, moral distress for nurses, inability for nurse to be honest with patient/family, family may question why patient is not getting better and become angry.

Todd et al. [36]	Exploratory mixed methods design	Acute care; 2 teaching hospitals, 17 Acute Care Nurses; Canada	Advocate: initiator, 76% used the term advocate. One nurse reported nurses argued with physician about a decision to place PEG where patient died soon after. Nurses do not always actively seek to be part of decision making process. Information broker: educator, teacher, provided information and answered questions. Liaison with physician or mediator. Nurses encouraged patients to talk to their physician and ask questions. Supporter: support provider.

Verhaeghe et al. [31]	Grounded theory	ICU; 1 University, 1 regional hospital, 22 family members; Belgium	Information broker: family identifies the nurse who gives adequate information. Some nurses give information that leads to false hope. For example, if the nurses says his BP is stable, family may interpret that as good, when really the patient is doing much worse. Supporter: caring, telling little details about daily care or patient. Advocate: inform family about how the patient's condition is progressing, are things “moving in the right direction.” More than just facts, but interpretation of facts. Outcomes: families may misinterpret facts as a good prognostic sign. The way the information is given to the family affects their ability to come to terms with the ICU experience and dying process.

Viney [26]	Phenomenology	ICU; 5 physicians 5 nurses; UK	Information broker: relay information between family and physicians. Speak to physician on behalf of family, “put in two pennyworth.” Supporter: empathy, prepare family for withdrawal. Advocate: game playing, indirectly influencing the physician. Not actively involved in the decision making process

Weber et al. [50]	Grounded theory	ICU; 3 ICUs in 1 hospital, 10 Physicians 23 Nurses; US	Advocate: advocate to physicians and to family. Use results of prognostic tool to initiate EOL discussions with physician and family.

Wise [51]	Mixed methods	Acute Care; 3 hospitals, 1 6 nurses (Phase 1), 100 nurses (Phase 2); US (Florida)	Advocate: intervene with physician, explain things to family, help them see futility, start EOL discussions. Get help and advice from more confident or experienced nurses. Nurses reported “standing up” to the physicians to advocate for patients.

Zaforteza et al. [41]	Qualitative	ICU; 14 observations, 6 nurse interviews; Spain	Information broker: gave meaningless information, such as “he/she has had a good night,” or “he/she has slept more or less.” Supporter: nurses ignored family members, did not introduce themselves but focused on technical activities. Nurses did not try to establish a relationship with the families. Advocate: did not give any interpretations about how patient is doing.

Zomorodi and Lynn [43]	Qualitative descriptive	ICU; 9 nurses; US	Information broker: ask physician to speak to the family. Supporter: calm, flexible, communicate with others well, pain and symptom management. Nurse sets own opinion aside and allows family to make decision. When decision made to withdraw, nurse takes a step back to allow family time with patient. One nurse described allowing a family member to get into the bed and lay down beside a dying relative. Advocate: when asked what would you do, answer “what do you think the patient would want.” Talk to physician and be frank about assessment that care is futile and should change direction. Be assertive with physicians.

**Table 2:** Summary of roles, strategies, and outcomes.

Information broker	Supporter	Advocate	Patient and family outcomes
Give information to physicians	Build trust	Advocate to physicians	Accept that patient is dying
(i) Patient and family preferences	(i) Introduce self and oncoming nurse	(i) Speak out in meetings	(i) Prepare
(ii) Emotional readiness	(ii) Practical needs	(ii) Question or coach	(ii) Help let go
(iii) Clinical condition of patient	(iii) Provide details about patient and daily care	(iii) Plant seeds	
	(iv) Accept decisions	(iv) Time discussions around physician seen as most open	
	(v) Explain equipment		
	(vi) Willing to talk		
	(vii) Rituals		
	(viii) Storytelling and life review		
	(ix) Help maintain hope		
	(x) Prepare for bad news		
	(xi) Assess readiness		
	(xii) Negative: ignore family and focus on technical details		

Give information to family	Empathy	Advocate to family	Make better decisions
(i) Educate about disease process	(i) Emotional support	(i) Give clear information	(i) Get the truth from nurses
(ii) Explain equipment	(ii) Acknowledge feelings	(ii) Interpret information	(ii) Understanding of prognosis
(iii) Translate/interpret medical terms	(iii) Take time to listen	(iii) Explore goals	(iii) Trusting relationships, allowed family to ask more questions
(iv) Clarify	(iv) Support physicians as well as family members	(iv) Explain implications of decisions	(iv) Move along in decision making process
(v) Educate	(v) Allow family time to process information	(v) Encourage to consider what patient would want	(v) Good death
(vi) Give information only without interpretation		(vi) Describe how patient is responding to treatment	(vi) Fear that families carry burden of guilt
(vii) Provide meaningless information		(vii) Explain prognosis	(vii) Satisfaction with care
		(viii) Blunt at times	
		(ix) Tell family patient is dying	
		(x) Sometimes vague and not involved	

Mediate		Extent of nursing advocacy:	
(i) Coordinate family meetings		(i) 75% actively involved in EOL decisions	
		(ii) 42%–54% discuss EOL decisions with patient or family members	
(ii) Consult other disciplines			
(iv) Request ethics consult			
(iii) Facilitate communication between family and medical team			
(iv) Ask physician to speak to family			
(v) Coach family in what to ask physicians			
